# Patriotism and the Nurse-Training Schools

**Published:** 1916-01-22

**Authors:** 


### PATRIOTISM AND THE NURSE-TRAINING SCHOOLS.*

### VI.—GUY'S HOSPITAL.

THE hospitals with large Nurses' Institutes attached were in a specially favourable position for responding to the patriotic call which echoed in every training school in August 1914. Guy's was among the very first to send off a large contingent, and throughout the whole period of the war Guy's nurses have done their part with brilliant success. A particular feature of this training school is the *esprit de corps* which binds the past and present members together in distant parts of the globe no less than in the grimy precincts of the hospital. The matron has been successful to a marked degree in keeping touch with those who are nursing the wounded abroad (each one, for instance, received a Christmas gift from the nursing staff), and has been instrumental in sending valuable assistance to the hospitals in localities to which they were attached, and more especially to No. 26 General Hospital, Etaples, where Miss Suart is at work.

Nearly a hundred members of the hospital staff and of the Nurses' Institute left their posts to take up work under the Admiralty and War Office. The list below of Guy's nurses, however, extends to nearly 300 names, and is even so not complete.

Out of this number Miss M. C. Corbishley and Miss M. J. Lyons, Q.A.I.M.N.S., and Miss V. Kiddle, Civil Hospital Reserve, have been awarded the Royal Red Cross. Miss E. C. Cheetham, Miss Suart, and Miss G. Allen, all in Q.A.I.M.N.S. or Reserve, and Miss E. Densham, British Red Cross Society, have had the honour of being mentioned in Sir John French's dispatches.

### WORKING ABROAD.

In addition to these, the following nurses trained at Guy's Hospital are at work in military hospitals abroad:—

*Q.A.I.M.N.S.*—M. Davis, A. B. Nunn, M. L. Potter, R. Rooke, H. Suart, G. Allen.

*Q.A.I.M.N.S. for India.*—M. E. Tippetts, P. Exshaw, A. R. Lowe, A. M. Hart.

*Q.A.R.N.N.S.*—D. Shewell, M. Noble, A. M. Shrewsbury.

*St. John Ambulance, British Red Cross, and other Hospitals.*—L. Wood, F. Hall, G. White, M. Gwilliam, M. Nation, F. Lee, R. Masters, N. Worsley, H. Green, K. Cadell, H. Bruce, E. Millidge, C. May, E. Fletcher, G. Fletcher, E. Ryman Smith.

*Q.A.I.M.N.S. Reserve.*—E. Jolley, F. Finnis, L. Mallandaine, N. Connolly, M. Colston, R. Macmorland, R. Leggoe, E. Batley, F. Brown, L. Tyers, C. Shann, W. Gillett, E. Bott, M. de Carteret, G. Corder, L. Fox, V. Lulham, E. Carter, E. Eaton, M. Spurway.

*Nursing Service of the Territorial Force.*—F. Edmonds, A. Coward, A. R. Cook, D. Fawcus, M. Healey, E. Ransford, M. Chittock, C. Todd, C. Dale, E. M. Leslie, W. Molesworth, A. Jolliffe, J. Lefebvre, F. Ford, C. Pearson, E. S. Sandeman.

*Civil Hospital Reserve.*—F. A. Spedding, M. Parsons, W. Evans, M. Dodson, I. Keeble, F. Davis, S. Surtees, B. Daniels, L. Robinson, M. Nelson, E. Quilter, W. Warner, G. Cosby, N. Hiles, E. Canter, D. Gower, F. Southcott, B. Twose, M. Goodchild, C. Barker, E. Squire, M. Mann, M. Owens, A. Prince, H. Sharwood, G. Waters, I. Ames, M. Beardshaw, M. Bourdillon, C. Druce, K. Mackenzie, D. King, V. Strutt, E. Gladstone, E. Macmanus, F. Hepburn, D. Wilson, E. Lear, M. Ripley, F. Broome, E. Bowdler, M. West, A. Sadleir, E. Adams, D. Grundy, R.R.C., K. Richardson, V. Tyler Cove, M. Tyndale, M. Maddison, M. Savage, K. Hayne, E. Johnson, A. Lithgow, E. Raven, S. Gibson, F. Brennand, M. Sawyer, G. Frank, M. Foxon, C. Pearse, A. Finlow, F. Morgan, C. Strange, L. Jenkins, E. Moriarty, A. McAra, A. Phillips, A. Daniels, M. Witchell, M. Douglas, M. Brown, E. Fraser, K. Hickman, E. Jickling, W. Todd, A. Beesley, M. Maclaren, M. Roussiano, H. Banbury, E. Orchard, L. Cornwell, M. Hilliard, M. Owen.

### ON DUTY AT HOME.

The following are some of the nurses trained at Guy's who are doing war work at home:—

*Q.A.I.M.N.S.*—E. Willes, M. Willes.

*Q.A.I.M.N.S. Reserve.*—E. F. Beloe, M. Carey, M. I. Wood.

*Civil Hospital Reserve.*—H. Vine, K. Vine, G. Stone.

*In Military Hospitals, etc.*—L. Anson, M. Atkey, E. M. Austin, C. Britton, M. Hay Bell, J. F. Ballantyne, F. E. Brown, E. Bishop, N. R. Bryan, E. M. Barber, M. Bryan, A. F. Boys, K. Blenkarn, D. Carey, E. M. Cooper, G. Cornell, A. Davidson, L. Denton, C. M. Densham, M. de Laveleye, S. Dottridge, A. M. Fletcher, M. Fraser, I. L. Edwards, K. Goule, E. M. Goss, L. Hodgson, W. Jones, C. Lyndon, A. Killpack, M. T. Morgan, E. B. Payne, A. von Pein, L. F. Wright, E. Wacher, E. Whittam, C. Krauth, A. S. Woodhead, A. M. Ward, Ferdinand, E. M. Latham, R. Groom, A. Gordon, E. K. Jewell, K. E. Finnemore, G. Field, E. Grant, A. M. Hooper, S. A. Hyland, M. H. Hobhouse, E. Horton, E. Hurlbatt, G. Harden, B. Hope, E. E. Heath, M. E. Jones, M. Johnson, A. Lloyd, W. Luckin Smith, G. Lulham, J. H. Lay, G. Leach, C. A. T. McKay, K. L. Morrall, A. M. Middleton, A. G. Macreath, M. Mansfield, F. Malkin, M. Marsh, M. A. Mumford, E. M. Newton, G. Osborne, M. E. O'Neill, E. O'Donnell, E. O'Rorke, M. O'Reilly, A. L. Phillips, Alice M. Phillips, E. M. Studdert, E. Stedman, D. Shepherd, F. E. Slack, A. du Sautoy, C. du Sautoy, E. M. Turner, K. E. G. Taylor, E. F. Tubbs, L. Turner, M. Vivian, M. E. Vanes, E. Windemer, E. Wood, G. Browning, F. M. Probert, R. S. Blyth, A. M. Blott, M. J. Sinclair, L. Purvis, R. E. Salter, G. E. Cannell, A. H. Maycock, E. Hope, G. Jenkins, M. B. West, S. Ellis, E. A. Stephens, L. Bedwell, E. Leedham, A. Bridges, I. Collie, M. Foley, E. S. Pace, J. Murray, F. J. Macdonald, M. A. Jones, E. I. Greaves, M. H. Self, A. F. Dean, G. Moss, E. de V. Fletcher, B. Poole.

* Previous articles appeared on October 23, October 30, November 6, November 20, and December 4, 1915.

